# Perceptions, facilitators and barriers of physical activity among people living with HIV: a qualitative study

**DOI:** 10.1186/s12889-023-15052-9

**Published:** 2023-02-18

**Authors:** Brenda Kitilya, Erica Sanga, George PrayGod, Bazil Baltazar Kavishe, Kia Ditlevsen, Robert Peck, Mette Frahm Olsen

**Affiliations:** 1 Mwanza Research Centre, National Institute for Medical Research, P.O BOX 1462, Mwanza, Tanzania; 2 Department of Food and Resource Economics, University of Copenhagen, Copenhagen, Denmark; 3 Weill Bugando School of Medicine, Mwanza, Tanzania; 4 Weill Cornell Medicine, New York, USA; 5 Department of Nutrition, Exercise and Sports, University of Copenhagen, Copenhagen, Denmark; 6 Department of Infectious Diseases, Rigshospitalet, Copenhagen, Denmark

**Keywords:** People living with HIV, Social ecological model, Exercise, Social-cultural attributes, Physical activity

## Abstract

**Background:**

People living with HIV (PLWH) have low levels of physical activity. Using the social ecological model to understand perceptions, facilitators and barriers of physical activity in this population is of importance for developing contextualised interventions to improve physical activity in PLWH.

**Method:**

This was a qualitative sub-study conducted between august and November 2019 as part of a cohort study on diabetes and associated complications in HIV infected in Mwanza, Tanzania. Sixteen in-depth interviews and three focus groups with nine participants in each were conducted. The interviews and focus groups were audio recorded, transcribed and translated into English. The social ecological model was considered during the coding and interpretation of the results. Transcripts were discussed, coded and analyzed using deductive content analysis.

**Results:**

Forty-three PLWH aged 23–61 years participated in this study. The findings showed that most PLWH perceived physical activity as beneficial to their health. However, their perceptions of physical activity were rooted within existing gender stereotypes and roles in the community. Running and playing football were perceived as activities for men while household chores activities were for women. Further, men were perceived to do more physical activity than women. For women, household chores and income-generating activities were perceived as sufficient physical activity. Social support and engagement of family members and friends in physical activity were reported as facilitators of physical activity. Reported barriers of physical activity were lack of time, money, availability of physical activity facilities and social support groups, and poor information on physical activity from health care providers in HIV clinics. Human-immunodeficiency virus (HIV) HIV infection was not perceived by PLWH as a barrier for doing physical activity but most family members did not support them to do physical activity, fearing that it might worsen their condition.

**Conclusion:**

The findings demonstrated differing perceptions, facilitators and barriers of physical activity among PLWH. Interventions addressing awareness, gender stereotypes and roles related to physical activity from individual to community level are needed. Supportive environment and infrastructures are needed to improve physical activity levels in PLWH in Tanzania.

## Background

Physical activity improves quality of life and reduces the risk of non-communicable disease (NCDs) [1, 2]. WHO guidelines recommend 150–300 minutes of moderate to vigorous intensity of physical activity per week to reduce the risk of NCDs as well as improve health and well-being [3]. Globally, 1 in 4 adults do not meet the global recommended levels of physical activity [4].

In Sub-Saharan Africa (SSA), where there is a high burden of human immunodeficiency virus (HIV) [5], physical activity may improve the quality of life of people living with HIV (PLWH) [6–8]. However, physical activity appears to be poor among PLWH with few studies reporting that PLWH spend ~ 75% of their time sedentary [9, 10]. Contributors to physical inactivity among PLWH include chronic inflammation, side effects of antiretroviral therapy (ART), poor nutrition, and other HIV–related co-morbidities [9, 11].

Besides biological drivers of reduced physical activity, studies have reported a number of behavioural and socio-cultural attributes which contribute to low level of physical activity among PLWH. These include lack of time, knowledge, motivation, concerns about physical appearance, tiredness from work, lack of financial resources to pay for physical activity facilities and lack of support from the family and community members [12–14].

The majority of studies assessing underlying factors contributing to low physical activity among PLWH adults have been conducted in older populations of high-income countries [15]. Studies included evaluations of participation and motivation in individuals or community-based physical activity programs [16–18]. Studies focusing on perceptions and facilitators have highlighted that PLWH perceive physical activity to be beneficial for their health but also find it challenging to be active [19]. In addition, these studies have reported that the facilitators and barriers of physical activity were related to psychological, physical, and social–environmental dimensions [19]. However, studies conducted in high-income countries cannot be extrapolated in SSA due to differences in societies, cultures, and health systems. Studies on HIV, social cultural issues, and physical activity in SSA are scarce [20], and this has been stipulated as a gap in a recent systematic review on physical activity and HIV in SSA [21].

Many theoretical and behavioural models have been used to understand and conceptualise factors influencing initiations, practices, and maintenance of physical activity [22]. Previous studies have used various theoretical models of behavioural change including the health belief model, trans-theoretical model and the social-cognitive model [23–25]. However, critics of these health-behavioural models have argued that changes focusing on individual’s lifestyle or behaviour including physical activity needs more than psychological–behaviour approach, since individuals make decisions and choices within a wider social-ecological context [26]. The theoretical social ecological model (SEM) shows the interplay of all levels including; interpersonal, intrapersonal, organizational and policy, illustrating how factors at one level influence factors at another level in behaviour [27]. The strength of the SEM model is that it provides a comprehensive understanding of the determinants of health behaviours and mechanism of change for each level [27, 28].

Previous studies have shown that cultural-sensitive behavioural theories such as the SEM model are useful in attempting to understand the factors which influence physical activity behaviour in vulnerable populations [15, 29]. In addition, other studies have used SEM to create and maintain interventions which facilitate communities to actively engage in physical activity [27–29]. Using qualitative study, we aimed to explore the perceptions, facilitators and barriers of physical activity among PLWH to better understand multifactorial influences of physical activity at individual, social and family, community and environmental levels.

## Method

### Study setting and design

This was a qualitative study conducted in Mwanza region in the North-Western Tanzania from August–November 2019 among PLWH. The study was nested within the cohort study, the “Chronic Infection, Co-morbidities and Diabetes in Africa (CICADA)” registered at http://clinicaltrials.gov as NCT03106480 [30]. Mwanza has a population of ~ 2.7 million and HIV prevalence of ~ 9.2% [31]. The common ethnic groups found in the region are Sukuma, Haya, Jita and Kerewe and the main income-generating activities in this region are petty trading, fishing, livestock keeping, farming and mining [31].

This study deployed the SEM adopted from McElroy and colleagues [27] to understand the underlying perceptions, facilitators and barriers of physical activity in PLWH. The original SEM incorporates intrapersonal (individual), interpersonal (social and family), community, organizational and public policy levels. For this study, we did not assess organizational and public policy level as it was out of our scope. The main focus was to understand factors influencing physical activity in the every day’s lives of PLWH. We analysed community level which also included environmental conditions together to describe the overlapping of influences on physical activity including existing physical activity promotional programs and the availability of public institutions which support or provide access to physical activity [32].

### Recruitment of participants

The participants were recruited from the CICADA cohort study, purposively selected to provide rich information on perceptions, opinions, social-cultural values and practices of physical activity in the community and at the individual level. The purposive selection process considered representation of sex, age group, occupation, marital status, living area and antiretroviral therapy (ART) status **(**Table 1**)** [33, 34]. The CICADA cohort study recruited PLWH from HIV clinics located in the public health facilities in the Nyamagana and Ilemela districts. Potential study participants were telephoned and invited by the first author to come at the National Institute for Medical Research clinic for more information about the study. Those interested were given a detailed written information sheet to read and an informed consent form to sign before being enrolled in the study. A total of fifty participants were invited, forty-three participants agreed to participate in the study while seven participants did not want to take part due to time constraints or being relocated from Mwanza.

**Table 1:** Characteristics of people living with HIV participating in in-depth interview and focus group discussions

Characteristics	Number of participants (n/%)
**Gender**	
Female	23 (53.5%)
Male	20 (46.5%)
**Age group (years)**	
18–25	3 (7.0%)
16–30	10 (23.2%
31–45	24 (55.8%)
46–65	6 (14%)
**Time since diagnosis of HIV (years)**	
1–3	33 (76.7%)
> 3	10 (23.3%)
**Marital status**	
Single	9 (20.9%)
Married	10 (23.3%)
Divorce/ Separated	18 (41.9%)
Widow/widower	6 (14.0%)
**Occupation**	
Business owner/Petty trader	30 (69.8%)
Employed	7 (16.3%)
Unemployed/housewives	6 (14%)

### Data collection, management and coding procedures

Data collection, management and coding were conducted in an iterative process informed by relevant guidelines to report qualitative research (Consolidated Criteria for Reporting Qualitative Studies checklist, COREQ) [35]. For credibility, we used two qualitative data collection methods. Sixteen in-depth interviews (interviews) were conducted to capture in-depth personal distinct, awareness, opinions and experiences of physical activity, whereas three focus-group discussions (focus groups) were used to capture shared common opinions, values and experiences within the communities on perceptions, facilitators and barriers of physical activity [36, 37]. We used different data collection methods to address specific dimensions of each objective. The final number of interviews and focus groups was based on when data saturation was reached [38].

The topic guides for the interviews and focus groups were developed in English and consisted of open-ended questions on perceptions, facilitators and barriers influencing physical activity [19, 28, 29, 32]. The topic guides were translated into Kiswahili and included questions about participants’ perceptions of the terms used to express “physical activity” and “exercise” (“Shughuli za Mwili” and “Mazoezi” respectively). These terms describe different concepts in Kiswahili. By definition, physical activity is defined as any bodily movement produced by skeletal muscles that result in energy expenditure in daily living and can be categorised from four domains: occupation, recreational sports, domestic, and transportation [39]. Exercise is defined as a subset of physical activity that is planned, structured, and repetitive with an objective of either improving or maintaining physical fitness [39]. However, in practice, the words are often used interchangeably [3], and this has previously created some challenges and misunderstandings in the interpretation of physical activity and exercise when used in other languages, cultures or contexts [4]. In our study, we used the two terms interchangeably throughout in interviews and focus groups. Questions about existing supportive or challenging circumstances and environment for physical activity practices, awareness, motivations, practices of physical activity, and whether these had changed after being diagnosed with HIV were included. The guides were pilot tested in three interviews to assess language, concept and content understanding of the questions and were then revised accordingly before they were used in the study.

BK and ES conducted all the interviews and focus groups in Kiswahili. BK and ES are female researchers at the National Institute for Medical Research with 10 years of experience and training in conducting qualitative studies. BK and ES worked together interchangeably as interviewer and note taker during the data collection process. The interviews and focus groups were conducted at the National Institute for Medical Research clinic where the participants attended their routine visits for the CICADA study. We ensured a conducive environment for interviews and focus groups (quiet rooms with adequate privacy) to allow freedom of expression. Each interview and focus group began with informal conversations with the intent of building rapport with participants. Throughout the interviews and discussions, we also conducted member checking by recapping of questions/responses with the respondents and this allowed validation of the responses. We also validated their responses during the data analysis and interpretation of results by making follow up with the participants through phone calls as recommended by Birt et al. [40] for trustworthiness. For focus groups, the first group was for men, second group was for women and third group included both. The two focus groups were conducted by sex category to avoid gender and power relations between men and women when responding to the questions. Interviews lasted about 30 min while focus groups lasted at least 45 min.

Interviews and focus groups were audio recorded using Sony tape recorder (IC recorder, ICD PX470, China) and fully transcribed and translated to English by an accredited research assistant independent of the researchers who collected the data.

Data were coded manually and analysed according to the principles of deductive content analysis [41]. Specifically, content analysis allowed us to condense and broaden the description of the physical activity phenomenon in PLWH, and the outcome of the analysis was described either in concepts or categories of factors influencing physical activity among PLWH [41]. First, the transcripts were thoroughly read. Based on the readings, the empirical material was categorised in the following major themes: *1) perceptions of physical activity, 2) facilitators of physical activity and 3) barriers of physical activi*ty. Secondly, following this initial categorisation of major themes, we identified sub-themes and sub-categories within each major theme (perceptions, facilitators and barriers) which was then coded as belonging to the individual level, the social and family level, or the community and environmental level as described in the SEM **(**Table 2**)**. The coding of perceptions, facilitators and barriers of physical activity at the individual and social and family level mainly used data from interviews, while the coding of themes at the community level used data from focus groups. In addition, participants’ quotes were used to illustrate the themes of perceptions, facilitators and barriers of physical activity at the individual, social and family, and community and environmental level.

**Table 2:** Perceptions, facilitators and barriers of physical activity people living with HIV according to the Social Ecological Model

Social ecological model levels	Unit of analysis/major themes	Sub-themes/factors
Intrapersonal (individual)	Perceptions of physical activity	Awareness and interpretation of physical activity
		Experiences and practices of physical activity
		Benefits of doing physical activity to health
	Facilitators	Motivation and reasons to do physical activity
	Barriers	Health concerns
		Lack of resources and infrastructure
		Time management
Interpersonal (social and family)	Perceptions	Family and friends’ opinions, experiences on physical activity to PLWH
	Facilitators	Emotional and physiological social support and interaction
	Barriers	Family and friends’ discouragements to do physical activity
		Fear of the disease/HIV-condition to worsen
Community / environmental	Perceptions	Attitudes and beliefs of physical activity
		Embedded social and cultural norms (stereotypes/gender roles) of physical activity
	Facilitators	Availability of resources and infrastructure to do physical activity
	Barriers	Lack of public health messages and groups for physical activity
		Little advice on physical activity from health care professionals
		Weather
		Personal identity and religious concerns
		Lack of public physical activity facilities / infrastructure

Data coding and interpretation was conducted by BK and ES separately and then compared. If there were any disagreements on the data coding and interpretation of themes, the two researchers would go through the transcripts and discuss the differences until consensus was reached.

### Ethical considerations

Ethical approval for the study was provided by the Medical Research Coordinating Committee of the National Institute for Medical Research with reference number NIMR/HQ/R. 8 alY ol. lX/ 2264 and Catholic University of Health and Allied Sciences Ethics Review Board CREC/415/2020. All eligible participants were informed of the study purpose and procedures including voluntary participation, and the right to withdraw at any time from the study procedures without any consequences. Participants provided a written informed consent prior to their enrolment to a member of the research team. Confidentiality and anonymity were maintained throughout the study.

## Results

### Participant’s characteristics

A total of forty-three participants above 18 years and on ART services for ≥1 year, participated in the sixteen interviews (eight men and eight women) and three focus groups with nine participants per focus group (nine men, nine women and four men/five women).

### Results overview

We found differing interpretations of physical activity and exercise which were based on specific physical activity practices rooted within the existing gender stereotypes and gender norms in the communities. Despite of the variation in the interpretation of physical activity, most PLWH perceived physical activity as beneficial for their health and were motivated to do it; if their family members and friends provided physical and emotional support that included doing physical activity together. Barriers of physical activity mentioned were time constraints, lack of social groups, lack of access to financial resources and facilities to be able to do physical activity. HIV-infection was not mentioned as a main barrier of physical activity but rather as a health concern from family members and friends thinking that physical activity could deteriorate their health. PLWH also reported that they hardly heard any public health messages regarding physical activity from health care professionals in HIV clinics and do not find any public health physical activity promotional messages displayed in the health facilities or community.

### Perceptions of physical activity

#### Individual level

##### Awareness and interpretation of the term ‘physical activity’

Participants revealed differing interpretation of the terms “*physical activity*” and “*exercise*”. The term “*physical activity*” was translated to (“*Shughuli za mwili*” in Swahili) which was perceived as an abstract concept; while the term “exercise” (“*mazoezi*” in Swahili) was perceived as a more commonly used term. Participants would generally describe physical activity practices with examples using the word “exercise”. Participants’ clearly identified physical activity with certain practices. Household chores and income-generating activities involving walking long distances were described as “physical activity”, In contrast “exercise” was perceived as recreational activities with the intention of improving physical strength or endurance. As reported by a participant:

*“Physical activity can mean work that you can do at home maybe to clean a house by mopping the floor or cultivating surrounding grass and exercise means maybe walking, rope skipping, going to the gym” [Interview-32 year old women].*

##### Experiences and practices of physical activity

Participants also perceived that income-generating activities such as petty trading; walking long distances selling fruits were sufficient physical activity for them. The commonly mentioned physical activity practices were walking, running, rope skipping and playing football which were reported to be easy to perform and suitable for many age groups. For example, as reported by a participant:

*“For me, the work I do requires a lot of physical activity and I also used to walk from town to Igoma as part of my exercise” [Interview-41 year old man].*

Some men perceived sexual intercourse was part of physical activity. This was reported in individual interviews as well as in participants’ focus groups on community perceptions of physical activity. For example, as explained by a participant:

*“Physical sexual intercourse with a woman is an exercise” [Interview-47 year old man].*

##### Perceived benefits of physical activity

PLWH perceived physical activity was important to their health, they believed by doing physical activity they will be able to improve their physical strength and health in general but also improve their personal appearance. As mentioned in an interview:

*“Physical exercise helps to keep you alive and keep the body in a good condition” [Interview-39 year old woman].*

#### Social and family level

##### Family members and friends’ opinions and experiences of physical activity

PLWH reported that family members and friends had low level of awareness, and poor physical activity practices. Despite this, some PLWH reported getting encouragement or company from friends who like exercise like running or walking. However, some respondents were unable to respond to this question because they lived alone and do not have many people around them. As reported by participant:

*“I don’t have groups or friends I like to live on my own.,I like listening to music if I have nothing to do” [Interview- 61 year old man].*

#### Community and environmental level

##### Perceptions of physical activity related to gender stereotypes and norms

Apart from the ‘individual’ and ‘social and family’ perceptions of physical activity, shared socio-cultural values in the community can also contribute to individual choices and behaviour of physical activity. In the focus groups, participants expressed perceptions of physical activity practices which were rooted within gender stereotypes and roles or norms in the community. The focus groups revealed that men perceived themselves and were seen by women as being more active than them. Out-door recreational physical activities were reported as more appropriate for men while household chores were identified as physical activity for women. As mentioned in a discussion:

*“(In) the society that surrounds me, men do more exercise like running though it’s just few of them. Women are only doing physical activity like household chores and take it as exercise” [Focus groups-34 year old woman].*

Women reported that household chores were sufficient physical activity for them, and that they did not need to do any more. As reported by PLWH in one of the focus group discussions:

*“Doing different activities help your body to become strong and resist against diseases. Myself, I fetch water and that is enough exercise” [Focus group-42 year old woman].*

##### Attitudes and beliefs on physical activity

Participants reported that other community members would associate certain types of exercise, such as weightlifting and exercise practiced in groups with criminal activities. This was reported in two focus groups and supported by the participants. As reported by a participant in a group discussion:

*“I remember I started exercising with my friend. We organized ourselves and then we started to exercise. After a short time, we inspired some other people and then formed a group of seven people. After that we found a place which was an unfinished house. Then we created local equipment for our gym, but some people in the community don't know the meaning of exercise. They think that exercise is a source of crime and terrorism, so they reported us to the street leaders. Eventually, (the street leader) came to our gym. He saw our place then took our gym equipment. He took the equipment with him thinking that exercise is the source of terrorism and crime in the community. After a few days he came back again. He realised we were just doing exercise, nothing else” [Focus group-27 year old man].*

### Facilitators of physical activity

#### Individual level

##### Motivation and reasons to do physical activity

Participants felt motivated to do physical activity because it restores their physical abilities and improves their health. Self-efficacy and self-esteem would also improve due to the ability to appear healthy and not be recognised as someone with HIV, as they reported:

*“What inspires me is, I want my body to be healthy and fit. Even if I am sitting among the people living with HIV, I cannot be recognised (as HIV-infected). I do it for my health, I want to stay well” [Interview-32 year old woman].*

#### Social and family level

##### Emotional and physiological social support and interaction

Family members and friends’ encouragements and participation in physical activity practices were reported as one of the main motivations for PLWH to do physical activity. Thus, the encouragement and participation of family members and friends are important facilitators of physical activity at the social level. As reported by a participant in an interview:

*“We play with the children in my area, like rope jumping. Sometimes we play netball. I even sometimes play football. I play with the youth there in our street” [Interview-32 year old woman].*

#### Community and environmental level

##### Availability of facilities

The presence and access to football pitches and open spaces in the community for physical activity was mentioned as one of the facilitators of physical activity. As reported by a participant:

*“In the community there is no gym, but we have football pitches and many people use it to run” [Focus groups-45 year old men].*

### Barriers of physical activity

#### Individual level

##### Time management

Participants commented that lack of time was one of the main barriers of physical activity. Participants did not have enough time to do physical activity because of work-related activities. As a participant explained in an interview:

*“I wish to walk more … I don’t have enough time … sometimes I find that I have a lot of work and there is no time to go walking” [Interview- 23 year old woman].*

##### Health concerns related to HIV

One participant feared physical activity would worsen their HIV disease leading to more deterioration of their heath rather than development of physical strength.

*“After diagnosis, I thought maybe doing hard work will make me weak, I thought like I will die and leave my children, but after some time I was okay” [Interview-45 year old woman].*

##### Lack of financial resources

Lack of resources or money to pay for access to fitness centres was also reported as a barrier of physical activity. As highlighted by a participant:

*“I won’t agree if they tell me to pay ten thousand (for access to a gym) while I earn five hundred thousands. It will be difficult … my income has to be in line with the gym costs” [Interview-61 year old man].*

#### Social and family level

##### Fear of disease to worsen from family members

Participants also reported that family members perceived physical activity practices to potentially worsen their HIV disease. Family members sometimes discouraged them from doing physical activity. This was mentioned only by few participants. As reported by one participant:

“*My mother advises me not (to exercise). She fears my condition” [Interview-30 year old woman].*

##### Lack of encouragement from family members

Family members discouraged physical activity due to lack of their time to participate in these physical activities. Immediate family members did not discuss or advise on any issues pertaining physical activity. As reported by the participant:

***“*** *Neither my husband nor my children tell me about doing any exercises” [Interview-36 year old woman].*

#### Community and environmental level

##### Lack of social programs to support physical activity

PLWH reported that lack of social groups participating in physical activity in their communities was a barrier to physical activity. In addition, both public health offices and HIV clinics failed to provide health messages to promote physical activity and that includes displays in the community on physical activity.

##### Little support and advice on physical activity from health care professionals

According to participants, health care professionals placed little emphasis on physical activity to PLWH at the health care centres. Hence, this was mentioned as a barrier since PLWH believed physical activity messages from health care professionals on physical activity would be like a reminder for them to do physical activity. The health care professionals mainly emphasise on maintaining a healthy diet, good hygiene and ART treatment adherence. As reported by a participant in a group discussion:

*“There is no place or groups for doing exercises. They (health professionals) never talk about exercise for sure” [Focus group-43 year old man].*

## Discussion

This study sought to explore perceptions, facilitators and barriers to physical activity among PLWH using the social ecological model. Our main findings of this study were as follows; physical activity was described and interpreted with language and cultural perspective that distinguishes physical activity from exercise and in practice physical activity was interpreted based on gender stereotypes and roles such as that household chores are for women while recreational exercise are for men. PLWH reported family members and friends’ advice and support motivates them to do physical activity apart from physical activity improving their health and wellbeing. Barriers of physical activity as reported by PLWH were lack of time, infrastructure and social groups, lack of financial resources for physical activity, and poor information sharing on physical activity from health professionals in the HIV clinics and community. Although HIV infection was not seen as the main barrier of physical activity among PLWH, family members were reluctant to encourage physical activity to their relatives because of fear that the HIV disease would worsen.

PLWH perceived and referred to physical activity as household chores activities, income-generating activities and other day-to-day activities as a sufficient level of physical activity while exercise was regarded as recreational or geared towards building physical strength. However, the majority of PLWH perceived that physical activity improves their physical strength, self-efficacy and health in general. Such perceptions on type, amount and practices of physical activity informs us about PLWH understanding of physical activity which may contribute to low levels of physical activity. Lack of awareness and misperceptions on the guidelines pertaining to physical activity inhibits physical activity [42, 43]. Physical activity programs raising awareness of physical activity in PLWH and their communities may lead to improvement of physical activity practices in this population and this has been shown in a study using health belief model that supervised exercise could change individual’s perceptions and knowledge of physical activity and the risks of NCDs [44] among the few studies in PLWH.

Research suggests that existing gender stereotypes and gender roles influence physical activity participation and choices across cultures and communities [45]. The present study also found that PLWH reported gender differences in their perceptions of physical activity. Certain physical activities were perceived as suitable for either men or women such as football for men and household chores for women. Also, it was perceived that men do more physical activity than women. Women perceived that they were doing less physical activity than men although they might have done quite a bit of physical activity through household chores. Women also expressed that household chores and income-generating activities were enough physical activity but they could also be enabled to engage in more leisure physical activity like men. Similar findings were reported in other studies on social construction of gender stereotypes and physical activity. The studies highlighted certain physical activities were perceived predominantly for boys and “less cool” for girls. Boys were perceived as more active while girls spend more time socialising [45–47]. However, other study have shown that participation in physical activity at an earlier age contributes to a more flexible attitude later on toward gender norms in physical activity [48]. Further, a systematic review conducted in SSA reported that the traditional gender stereotypes of women not engaging in out-door activities influenced the level of physical activity participation because out-door physical activity was perceived as “unfeminine” [49]. Our findings highlight the need for contextual gender-based interventions appropriate for PLWH addressing positively the existing gender stereotypes and roles in relation to physical activity, providing opportunity for women to be able to do sufficient physical activity.

In this study, we also observed that physiological social support and interactions from family members and friends were facilitators of physical activity in PLWH. Friends and family members play a major role in enhancing physical activity. This social support in physical activity has been widely studied and has been noted to be a strong facilitator of physical activity among individuals. In other context, studies have gone further by indicating clearly the directions and differences of social support from friends and family members that could influence the motivations and maintenance of physical activity practices [50–52]. Even though these studies have been conducted elsewhere and not in SSA, their results and our results show some potential of transferability theoretically to understand how social support from family members and friends may enhance physical activity practices. PLWH reported benefits resulting from doing physical activity such as increased physical strength, self-efficacy and improved health which motivated them to initiate and maintain physical activity [1, 53]. However, other studies have argued that the concerns of PLWH for health improvements and improved physical appearance are among the fundamental coping strategies to reduce HIV related stigma [28]. Overall, our findings provide evidence for the value of social support to PLWH to increase physical activity in their day to day living. Interventions directly emphasizing on improving social support to PLWH from family members and friends will aid in promoting physical activity in this vulnerable population.

Some of the main barriers to physical activity reported among PLWH were time availability, lack of facilities, infrastructure and social groups, poor support from family members, high costs for recreational activities at fitness centres, and health concerns due to HIV-infection. Similar results were reported in other studies conducted in high-income countries and most were conducted in women with HIV and older populations and have used SEM and a comparison group of HIV-uninfected individuals to explore barriers of physical activity [54, 55]. These studies described biological factors resulted from the HIV infection such as physical exertion, opportunistic infections, presence of bodily pain, and depression as barriers to physical activity. Social and cultural factors including family discouragements, time constraints and unfamiliarity with physical activity facilities and machines in fitness centres have also been reported as significant barriers to physical activity [13, 55–57]. On the contrary, cultural issues relating to religion, cultural identity, body image and physical appearance were not identified as barriers to physical activity in our study, unlike in other studies [54, 57]. Nevertheless, there some other studies which reported illness stereotypes where people with chronic diseases share concerns about physical activity as a risk for their health [58].. Thus, this was also reported in our study by family members and PLWH. Findings observed in this study on barriers, may inform development of appropriate interventions for PLWH to improve their physical activity practices in this context and reduce long-term effects of non-communicable diseases and few studies on health promotion interventions in other age populations have highlighted promising results when it comes to improvement of physical activity [59].

Lack of support and advice on physical activity from health care services was also found to be among the barriers in the promotion of physical activity. PLWH reported lack of education on physical activity at the HIV clinics from health care professionals. The Tanzanian government’s management and care of HIV & AIDS guideline has clearly stipulated that in PLWH who are stable on ART, physical activity should be recommended to patients [60]. On the other hand, the recommendations for physical activity available in this guidelines are not detailed and are merely mentioned as a sub-topic in relation to diet [60]. Therefore, in practice, it can easily be overlooked or not stressed enough by health care professionals. The Tanzanian guideline has been criticised due its lack of alignment with the local concerns for ART service delivery [61] and for physical activity, lack of detailed information on physical activity recommendations [62]. The findings of our study support the importance of incorporating physical activity promotional messages in the management of HIV.

### The strengths and limitations of the study

The strengths of this study includes the use of SEM to explore in-depth the concepts and context of physical activity including social and cultural factors that influence physical activity among PLWH in their day to day living and their communities [53, 63]. By understanding these factors, public health specialists and policy makers would be able to design appropriate contextual based interventions which will promote physical activity among PLWH. In addition, to our knowledge this is the first study to explore social and cultural factors that influence physical activity among PLWH in Tanzania, and one of few studies in SSA.

Our study employed qualitative research guideline known as COREQ [64] during its implementation, data collection and reporting of the results. Throughout, the study assessed in detail the components of rigour and trustworthiness (credibility, dependability, transferability and conformability). The study used two different data collection methods, interviews and focus groups to address all the dimensions of the specific objective and the findings could be transferable to other settings, hence, this weights the strength of this study. In addition, the study was implemented by experienced qualitative researchers strictly following appropriate guidelines in all the procedures (selection of respondents, data collection and processing, analysis, interpretation and reporting of results).

One of the limitations of this study is that it has been conducted in only one city of Tanzania and thus, the results may not be transferrable to all settings or communities in Tanzania at large. In addition, some degree of interviewer bias and participant selection bias was unavoidable as the participants who agreed to be interviewed, were involved because they had time and interest to participate. We have chosen to structure our findings at the levels of the SEM model, but we acknowledge that several of the facilitators and barriers of physical activity are cross-cutting and at times do not present at a specific level in the everyday lives of PLWH. Finally, our sampling framework was limited to PLWH since our objective was to investigate the factors that influence PLWH and their physical activity practices. For future studies, we propose health workers and other members from non-government officials who work with PLWH to be involved in these studies to enrich additional information on perceptions and physical activity among PLWH, at the community and for policy implications level.

## Conclusions

We found there are differing interpretations of physical activity and exercise and that physical activity practices are rooted within existing gender stereotypes and roles in the communities. Physical activity related to household chores was for women and exercise or outdoor activities were for men. PLWH felt motivated to do physical activity because of the encouragements from family members and friends and because of perceived health benefits which included physical strength, improved health, and not looking like a person living with HIV. However, reported barriers to physical activity included lack of time, limited awareness, health concerns related to HIV-infection, lack of support from family and friends, lack of resources to pay for access to fitness facilities, lack of social groups for physical activity and lack of facilities. Further, existing gaps in implementing the management and care of PLWH guidelines in relation to physical activity was also observed as a barrier to physical activity as reported by PLWH. HIV-infection and other health-related outcomes were not mentioned as the main barriers by the PLWH but rather as a health concern from family members and friends for PLWH if they do physical activity. The main barriers were social-cultural issues which were the mirror of the facilitators of physical activity. The use of SEM model has allowed to us to identify and understand different factors at different levels (individual, social and family, and community and environmental) that influence physical activity among PLWH.

### Implication for policy and practice

This study was undertaken partly to inform future interventions or strategies to understand the individual, socio-cultural issues related to physical activity practices among PLWH and their communities. Development of new strategies to promote physical activity must address issues related to awareness and emphasise on commonly known practices of physical activity in relation to WHO recommendations. Further, involvement of family and friends to provide support emotionally and physiologically to PLWH on physical activity would help to improve physical activity among PLWH.

Interventions are needed to address the elements of gender stereotypes and roles, attitudes and beliefs related to physical activity that exist in the community and specifically to promote physical activity in women.

In addition, these results will also support development of new physical activity public health promotional messages, and to be displayed in healthcare facilities, public institutions and for health care professionals to share adequate information on physical activity to PLWH. Building of supportive environments including facilities or infrastructures for physical activity is needed in the communities to promote physical activity in PLWH.

## Data Availability

The datasets use/or analysed during the current study are available from the corresponding author on reasonable request.
